# Lesson learned from the COVID-19 pandemic: toddlers learn earlier to read emotions with face masks

**DOI:** 10.3389/fpsyg.2024.1386937

**Published:** 2024-07-03

**Authors:** Monica Gori, Lucia Schiatti, Monica Faggioni, Maria Bianca Amadeo

**Affiliations:** ^1^Unit for Visually Impaired People (U-VIP), Istituto Italiano di Tecnologia, Genova, Italy; ^2^La rotonda dei bambini, Scuola paritaria della coop. S.a.b.a., Genova, Italy

**Keywords:** emotion inference, facial configurations, social development, COVID-19, face masks

## Abstract

In a prior study we demonstrated that the presence of face masks impairs the human capability of accurately inferring emotions conveyed through facial expressions, at all ages. The degree of impairment posed by face covering was notably more pronounced in children aged between three and five years old. In the current study, we conducted the same test as a follow-up after one year from the onset of the COVID-19 pandemic, when the requirement of wearing face masks was holding in almost all circumstances of everyday life when social interactions occur. The results indicate a noteworthy improvement in recognizing facial expressions with face masks among children aged three to five, compared to the pre-pandemic settings. These findings hold a significant importance, suggesting that toddlers effectively mitigated the social challenges associated with masks use: they overcame initial environmental limitations, improving their capability to interpret facial expressions even in the absence of visual cues from the lower part of the face.

## Introduction

A deep understanding of emotions is paramount to establish effective social interactions. In particular, interpreting facial expressions plays a foundational role during the social development of children, as they learn to engage with others (Denham et al., 2014). While the processing of emotions is inherently present in infants, early childhood emerges as a pivotal period for the development of emotional understanding and processing (Denham et al., 2003). The trajectory of emotional development extends continuously from infancy to adulthood, marked by ongoing changes as individuals navigate the expanding capacity and complexity of their social environments (Herba and Phillips, 2004; Tonks et al., 2007; Barrett et al., 2019).

**Figure 1:**
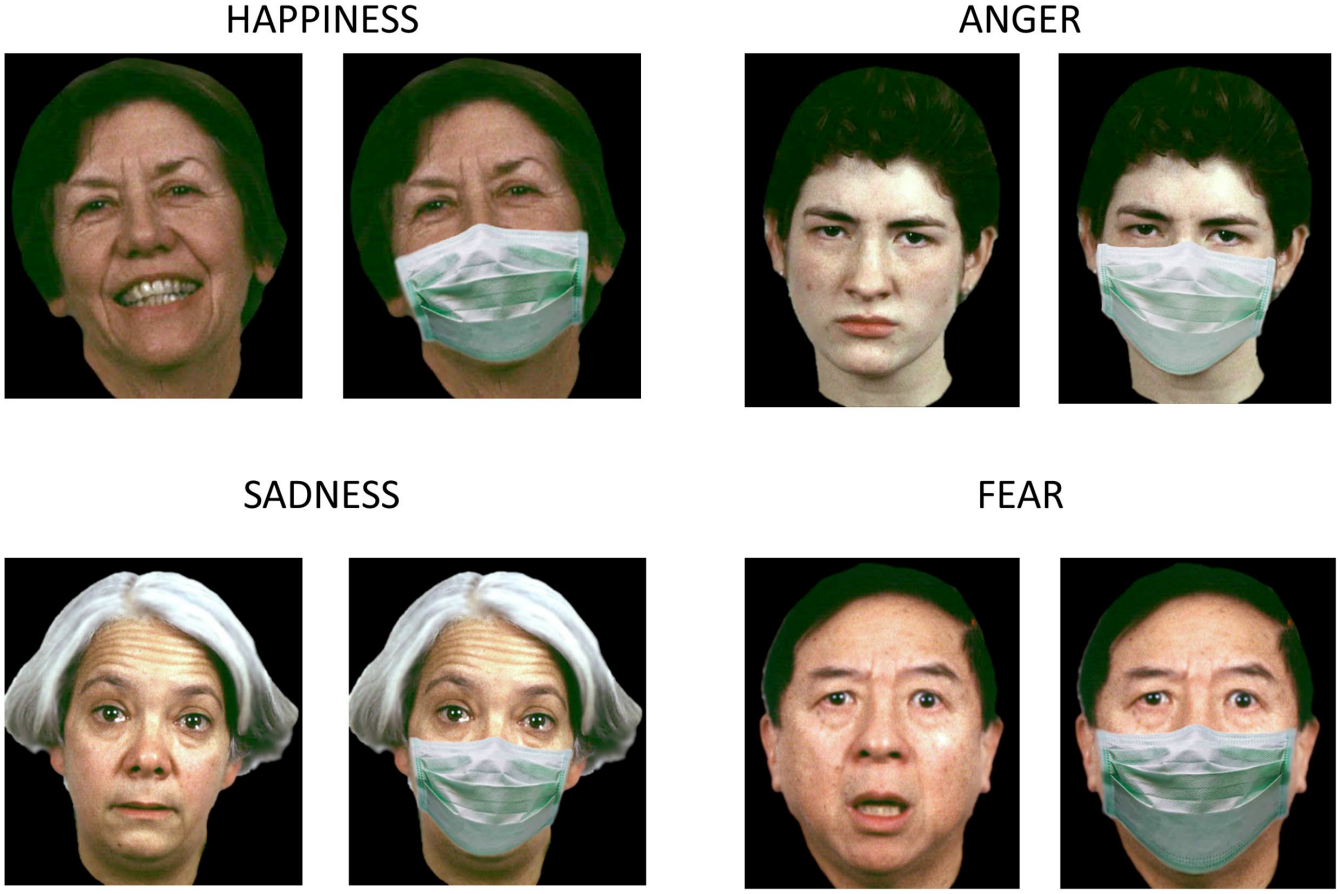
Examples of facial expressions with and without face masks for happiness, anger, sadness, and fear. Original face images were obtained with permission from the ER-40 color emotional stimuli public database (Gur et al., 2002; Pinkham et al., 2008). With permission from Amadeo et al. (2022).

**Figure 2:**
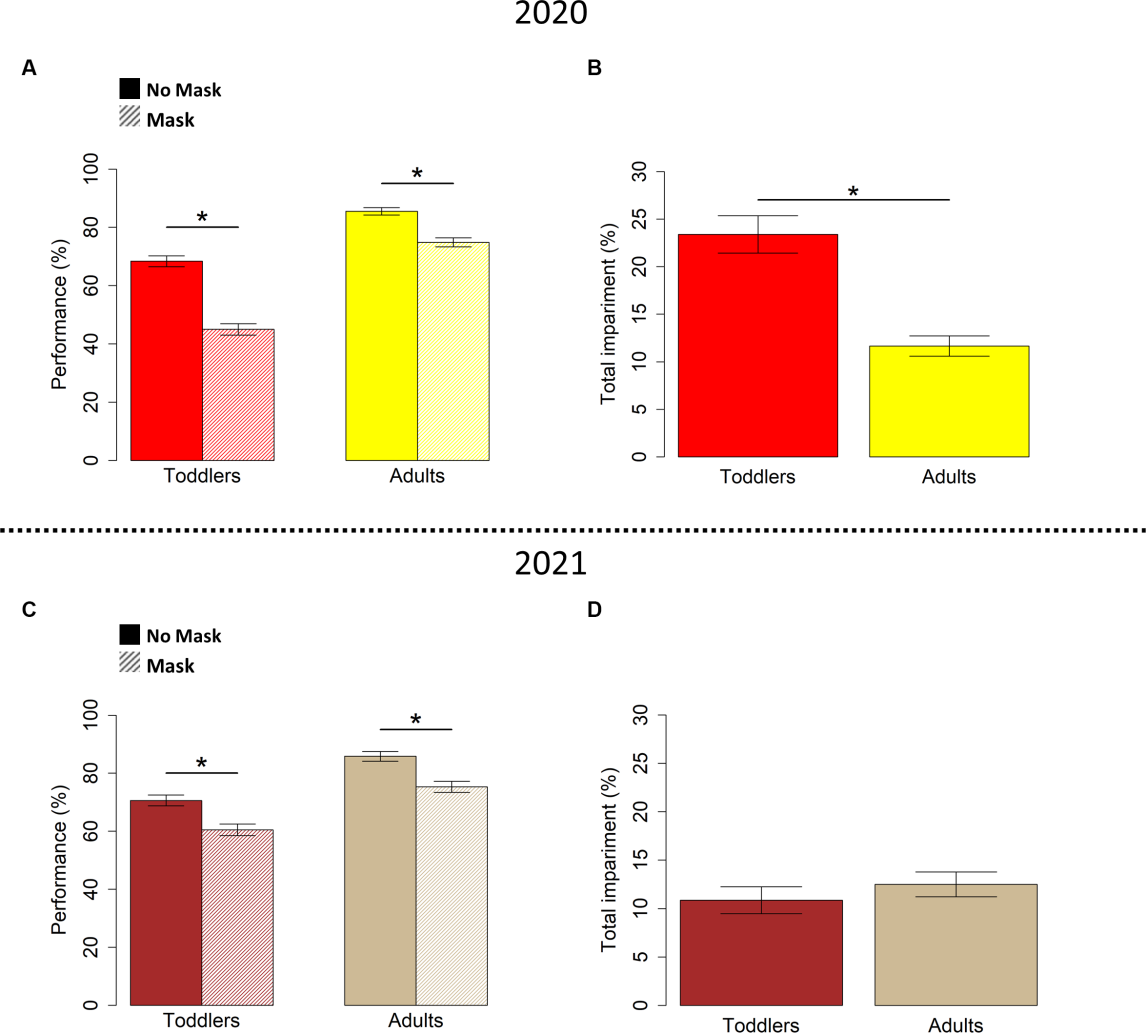
Impact of face masks on emotion recognition at the onset of the COVID-19 pandemic (top panel) and one year later (bottom panel). Top panel: results readapted from previous paper (Gori et al., 2021) investigating emotion recognition with face masks at the onset of the COVID-19 pandemic. **(A)** Percentage of correct responses in emotion recognition from facial configurations without and with face masks in toddlers and adults. **(B)** Percentage of impairment due to masks in toddlers and adults. Bottom panel: results about emotion recognition with face masks one year after the onset of the COVID-19 pandemic. **(C)** Percentage of correct responses in emotion recognition from facial configurations without and with the masks in toddlers and adults. **(D)** Percentage of impairment due to masks in toddlers and adults. Standard Error of the Mean (SEM) is reported. The stars indicate a significant difference between the groups (*p* < 0.001).

Previous research demonstrated that the immediate perception of others’ emotions often relies on the observation of facial movements (Grundmann et al., 2021; Marini et al., 2021; Pavlova and Sokolov, 2022). Between 2020 and 2022, the spread of the COVID-19 emergency introduced a natural impediment to the processing of faces. The widespread use of face masks forced people to read others’ faces without the visual cues we normally gather from the mouth and the lower part of the face, affecting several circumstances of everyday life where social interaction occurs (e.g., at school). As so, the COVID-19 pandemic represented a distinctive opportunity to investigate how the ability to infer emotions from facial expressions evolves in children and adults when both are forced to interact with individuals wearing face masks. A captivating inquiry we delved into centered around how the ability to recognize facial expressions is impaired in toddlers, children and adults when a portion of the face is covered by surgical face masks (Gori et al., 2021). Our hypothesis, substantiated by experimental data, posited that the recognition of emotions from facial expressions was influenced by the presence of masks. To test such a hypothesis, we administered a forced choice verbal-response test based on selecting an emotion’s label to describe static pictures of human facial expressions, related to five specific emotions (angry, sad, happy, fearful, and neutral). The test was modified to include a condition where original stimuli showing facial expressions were covered by surgical face masks (see Figure 1). We collected answers from subjects of three different age groups: adults, children aged 6–8 years old, and toddlers aged 3–5 years old. Our results demonstrated that the impact of face masks on facial expressions’ recognition was stronger in toddlers, whose developmental trajectory in deciphering emotions from facial expressions was still ongoing, making them particularly susceptible to the presence of face masks (see Figure 2, top panel). On the other hand, the decrease of performance in reading masked versus unmasked emotions was similar in children and adults. In the last few years, several studies investigated the effect of face masks on the recognition of facial expressions and emotions’ inference on both adults and children (Pavlova et al., 2023). Although not all consequences of wearing face masks on perception, social cognition and communication are fully understood, a consistent body of research showed that difficulties in reading covered faces are cross-cultural, gender- and age-specific (Pavlova and Sokolov, 2022), and they vary based on individual differences and personality (McCrackin et al., 2022). There is a general agreement on the fact that face masks hamper facial affect recognition (Rinck et al., 2022; Ventura et al., 2023), even without compromising the capability of inferring basic emotions.

The COVID-19 pandemic also provided a chance to deepen into the mechanisms of human learning and adaptation in case of limited social interactions and prolonged changes in the availability of the information we can gather from facial expressions. Attempts to assess whether a learning and/or habituation process occurred in the capability of inferring emotions from facial expressions wearing face masks were already made. In particular, one study (Carbon et al., 2022) compared emotional expressions’ recognition (angry, disgusted, fearful, happy, sad, and neutral) from data collected on different samples of adults in Germany, on May 2020 and July 2021 respectively, and demonstrated no improvement in performance, both with and without face masks. Anyhow, no data from children younger than 18 years old were collected, and among existing literature there is still a lack of works on the effects of COVID-19 restrictions on developmental processes. In accordance with our previous study (Gori et al., 2021), existing findings show that school-aged children are still able to make inferences about emotions from masked faces, similarly to adults (Ruba and Pollak, 2020; Miyazaki et al., 2022). On the other hand, face masks significantly affect emotion recognition skills in pre-school children, with masked angry faces more easily recognized than other emotions (Bourke et al., 2023). Carnevali et al. (2022) presented a state-of-the-art framework of behavioral studies investigating face processing in early infancy (first 3 years of life), pointing out the importance of evaluating how face covering affects face reading during early development and sensitive periods.

Recognizing the inherently adaptable nature of the brain, especially in developmental stages (Kolb et al., 2013), here we explored the effects of long-term exposure to masked faces on toddlers’ capability of recognizing emotion-related facial expressions from masked faces. Indeed, pre-school children presented the higher impairment according to both existing literature and our previous results. We conducted a follow-up study starting on March 2021, i.e., around one year later than our initial investigation, which took place at the onset of the crisis (i.e., March 2020). During the initial study (Gori et al., 2021), both children and adults were experiencing the presence of face masks for the first time, requiring an effort to infer emotions from facial expressions where the lower part of the face was covered. This was particularly true for toddlers aged between three and five years old. In this follow-up study, children and adults had experienced face masks for about one year, making it a familiar condition during interaction with other people. The comparison between these two conditions allowed us to investigate the impact of experience and exposure to face masks on toddlers’ capability to infer emotions from partially occluded facial features. Our overarching hypothesis posited that if the massive exposure to masked faces prompted adaptation and learning over time, toddlers entering the 3–5 years old age range one year after the onset of the COVID-19 pandemic should exhibit an improved performance in facial expressions’ recognition of masked faces, potentially approaching the proficiency of adults, compared to toddlers who grew up in pre-pandemic conditions.

## Methods

To test our hypothesis, we replicated the experimental protocol outlined in our previous study (Gori et al., 2021) one year after the onset of the COVID-19 pandemic in Italy. Building upon prior findings indicating a specific challenge in recognizing emotions from facial expressions with face masks among toddlers aged 3 to 5 years old, we specifically targeted this age group. To ensure a sample of participants precisely matching the age criteria, we recruited a new set of 70 participants distinct from those involved in our previous study. The final sample included 40 toddlers (i.e., pre-school age) from 3 to 5 years old (mean age: 4.3 ± 0.7 years old), and 30 adults from 18 to 30 years old (mean age: 41.7 ± 11.4 years old). All participants were native Italian speakers. The study was approved by the local ethics authority (Comitato Etico, ASL3 Genovese, Italy) and an informed consent was obtained from the subjects or their parents before participating to the test.

Data collection lasted approximately six months, starting in March 2021, precisely one year after the first lockdown in Italy. During the year following the onset of the COVID-19 pandemic, in Italy face masks were mandatory for individuals aged above six in enclosed spaces, as well as in open settings where physical distancing was impractical. Kindergartens were closed in March 2020, and reopening starting from September of the same year. Since then, they have remained predominantly operational, with intermittent closures based on the trajectory of the pandemic and localized outbreaks. The kindergarten staff were required to wear face masks, while masks were optional for children. The experimental design and procedure were identical to our previous study (Gori et al., 2021; Amadeo et al., 2022; Escelsior et al., 2022). To assess emotions’ recognition from facial expressions we used a standardized verbal-response test based on selecting an emotion’s label (out of a forced-choice selection) to describe static images of posed human facial expressions. Stimuli were derived from the ER-40 color emotional stimuli database (Gur et al., 2002; Pinkham et al., 2008) developed for the validated ER-40 test for facial emotion recognition (Kohler et al., 2004; Carter et al., 2009). We generated the masked condition by adding a realistic face mask on original images (see Figure 1). The test was administered through an internet-based questionnaire presented via smartphones. Participants were required to label the emotion by choosing one out of five possible options (happy, sad, fearful, angry, neutral) from images of adult facial expressions with and without face masks. The task was structured in sequential blocks, first showing a set of pictures of people wearing face masks, followed by a block of mask-free images. For each block, four instances of each emotion (happy, sad, fearful, angry and neutral) were shown, resulting in 20 pictures of adult faces. The four non-neutral emotions included two levels of intensity (mild and extreme). Emotions were presented in a randomized order. Toddlers completed the questionnaire either at home or at kindergarten, receiving guidance from a caregiver or an experimenter. The reference person read the question to the child, as well as the multiple-choice options, while showing the current image, and then selected the child’s choice. Despite we could not ensure complete control during test administration at home, specific written instructions were provided to caregivers as in our previous experiment. No time limits were imposed to provide an answer.

All analyses were conducted using R (R Core Team, 2013). During the data analysis, the performance was calculated as a percentage of correct answers, both on stimuli with and without face masks. Furthermore, the overall impairment due to the mask’s presence was calculated as the percentage difference between correct answers with and without face masks. First, normality assumption of data was tested for performance and impairment with Shapiro–Wilk normality tests. Wilcoxon tests were then conducted to statistically compare the performance in each condition (Mask, NoMask) and age group (i.e., Toddlers, Adults) to chance level (i.e., 20%). Subsequently, performance was analyzed with a permutation-based analysis of variance (ANOVA) considering the condition (i.e., Mask, NoMask) as within-subject factor and the age groups (i.e., Toddlers, Adults) as between-subject factor [function aovp of pakage lmPerm (Wheeler and Torchiano, 2010)]. Initially, we considered also the type of emotion (i.e., happy, sad, fearful, angry, and neutral) as a within-subject factor in the ANOVA, but since we found no significant effect, we removed it from the main analyses. Based on our previous findings showing that the level of emotion’s intensity (i.e., mild, extreme) had no effect on the ability to recognize emotions in children and adults (Gori et al., 2021), we disregarded this factor in the current analyses too. *Post-hoc* analyses were conducted with permutation-based *t*-tests [function perm.t.test of pakage Deducer (Fellows, 2012)]. The statistical significance of the impairment was analyzed by comparing groups (i.e., Toddlers, Adults) with a permutation-based *t*-test. All results were corrected for multiple comparisons using Bonferroni correction.

## Results

Results demonstrate that the use of face masks affects the capability of inferring emotions from facial expressions at all ages, and that the overall impairment for toddlers decreased from 23.4% [based on data from the original study (Gori et al., 2021), Figure 2B] to 10.9% after one year of exposure to face masks (Figure 2D). Indeed, the impairment of toddlers in recognizing emotions from facial expressions with face masks became comparable to that of adults (around 11% both in the original study and in the current follow-up study).

We did not find a normal distribution of performance (for adults, with mask: *W* = 0.8, *p* < 0.001; for adults, without mask: *W* = 0.7, *p* < 0.001; for toddlers, with mask: *W* = 0.9, *p* < 0.001; for toddlers without masks: *W* = 0.9, *p* < 0.001), as well as of impairment (for adults: *W* = 0.8, *p* < 0.001; for toddlers: *W* = 0.8, *p* < 0.001).

In line with data from the original study (Gori et al., 2021), we confirmed that the ability of inferring emotions from facial configurations is significantly above-chance level (20%) for toddlers and adults both without face masks (for toddlers: *V* = 820, *p* < 0.001; for adults: *V* = 465, *p* < 0.001) and with face masks (for toddlers: *V* = 780, *p* < 0.001; for adults: *V* = 465, *p* < 0.001).

The permutation-based ANOVA with percentage of correct responses as dependent variable revealed a significant main effect of both the group (*F*_1,68_ = 32.6, *p* < 0.001, ges = 0.3), and the condition (*F*_1,68_ = 76.3, *p* < 0.001, ges = 0.5). This indicates that adults preformed overall better than toddlers, and both groups performed better without face masks. However, contrarily to our previous finding (Figure 2A), the interaction between group and condition was not significant (*F*_1,68_ = 0.03, *p* = 0.9, ges = 0.03). The lack of this interaction supports the hypothesis that the presence of masks impacts the performance of toddlers and adults in a similar vein. The permutation-based ANOVA investigating the interaction between group, condition and type of emotion was not significant (*F*_4,544_ = 0.7, *p* = 0.6, ges < 0.01), suggesting no effect of emotional content on the results. In other words, adults present an overall better performance, and the presence of masks is associated to a decrease of performance independently of age and emotion type (Figure 2C). The similar influence of face masks on emotion recognition for both toddlers and adults emerges also by analyses on the impairment associated with mask’s presence. Indeed, we found no significant difference between the impairment of toddlers and adults (*t* = 0.16, *p* = 0.9) (Figure 2D).

## Discussion

In this study, we explored how the ability to recognize emotions from facial expressions with face masks changed in children aged between 3 and 5 years old as well as in adults after one year of exposure to masked faces due to the COVID-19 pandemic.

### General better performance of adults

Adults performed overall better than young children. Building on previous findings (Ruba and Pollak, 2020), adults exhibited notably higher performance even in the presence of face masks compared to toddlers, with correct response rates exceeding 70%. This suggests that adults found it relatively easy to infer others’ emotions despite the presence of face masks. Neurophysiological perspectives, as outlined by Thomas et al. (2001), describe age-related changes in emotion inference between children and adults, attributing them to the development of advanced cognitive skills and increased efficiency in pre-frontal neural structures. In agreement with existing literature (Chronaki et al., 2015), our study confirmed that toddlers exhibit lower reliability than adults in labeling emotions from facial expressions without masks, although their performance largely surpassed chance levels (Gori et al., 2021).

### Lack of stronger interference of face masks on emotion inference during the development stage

Participants of all ages encountered increased challenges in recognizing emotions when face masks were present. However, a key finding of this research is that, in contrast to our previous data (Gori et al., 2021), after at least one year of exposure to face masks in everyday life, developmental differences in impairment due to face masks between toddlers and adults were no longer holding, i.e., both groups showed a similar drop in performance when inferring emotions from facial expressions with face masks. In the original study, face masks affected emotion understanding across all age groups, but with a pronounced impact on toddlers. Based on our new data, children growing in an environment where face masks were pervasively present, showed a diminished effect, and their performance was no longer disproportionately influenced by the presence of a mask compared to adults. This result is in line with Giordano et al. (2022), showing that an enhanced ability of children aged 3–5 years old to successfully identify emotions in masked faces is correlated with higher exposure to adults wearing masks and group care. Although it is known that a perceptual narrowing in face processing occurs during the first year of life, leading to a specialization in the processing of adult faces compared to child faces (Macchi Cassia et al., 2014; Kobayashi et al., 2018), it is unlikely that this aspect affects the present results. Indeed, our study focuses on children older than 3 years old, while the perceptual narrowing toward adult faces happens between 3 and 9 months of life. Furthermore, both the previous and the current study used images of adult faces as stimuli, thus playing in favor of children ability to infer emotions. Moreover, legislation in Italy required the mandatory use of masks only for individuals older than six years old, driving to the assumption that toddlers mostly experienced masked faces when interacting with adults rather than with other children. The lack of improvement in adults is in line with other research showing that reading emotions from faces with and without masks is relatively independent of extended exposure to masked faces for healthy individuals aged between 18 and 64 years old (Carbon et al., 2022).

### Learning to infer emotions from facial expressions with masks during the development stage

Inferring emotions from facial features plays a pivotal role in the development of children’s emotional processing and social competence. Ongoing developmental shifts persist from infancy to adulthood, and the ability to infer emotions from eye movements and speech does not stabilize until middle childhood and adolescence (Herba et al., 2006). Research examining the neural substrates associated with the observation of diverse facial movements aligns with behavioral findings, indicating that the processes of emotional reasoning do not reach adult-like levels until early adolescence (Batty and Taylor, 2006). In addition, our classifications of emotions are not fixed; they heavily rely on the types and frequencies of facial movements to which we are exposed (Plate et al., 2019) and children progressively improve their understanding of facial emotional cues through positive interpersonal relationships over time (Denham, 1998). Consequently, during a crucial period for emotional learning, young children are likely to be influenced by a massive exposure to individuals wearing face masks.

Our earlier findings suggested that the occluded access to facial configurations due to the use of face masks during the COVID-19 pandemic could negatively impact the development of emotional inference and the acquisition of social interaction skills in toddlers (Gori et al., 2021). This issue gained attention both at a social and institutional level. During the pandemic, the World Health Organization (WHO) and UNICEF provided guidelines for decision-makers and authorities about the use of face masks when interacting with children (World Health Organization, 2020). They generally discouraged the exposure to face masks for children up to five years old due to the attainment of significant developmental milestones at that age (Coppola, 2014). Our present findings offer a positive perspective about these concerns, showing that some of the potential risks might be mitigated by learning and adaptation processes.

Crucially, our first experiment was conducted at the onset of the COVID-19 emergency (within two weeks after the first lockdown phase in Italy), during which all participants experienced facial expressions with masks in their everyday life for the first time, and they mostly spent time at home. We can assume that the performance at this stage was unaffected by prior exposure or experience with face masks. By conducting the same study after one year of COVID-19 pandemic and its consequent safety regulations, we assumed that exposure to masked faces has increased significantly, as people began to interact more frequently with individuals wearing masks. From the decreased impairment in reading masked faces that we found in pre-school children, it is reasonable to conclude that the continuous and prolonged exposure to face masks led to an enhanced ability of toddlers to recognize emotions from facial expressions with face masks. One possibility to explain our results is that the one year-long exposure to face masks in various everyday social and educational settings likely allowed young children to develop other strategies, relying on alternative (e.g., contextual) cues to infer emotions. Another explanation of our results could be provided by an acceleration of the brain processes leading to a more robust capability of inferring emotions even from the eyes’ region only, as we observe in adults (Chung and Thomson, 1995; De Sonneville et al., 2022). We speculate that this improvement in face processing might be linked to the plasticity of the developing brain of children. Indeed, brain plasticity could have effectively supported the learning process allowing to utilize alternative cues associated with emotional reasoning, as well as an acceleration in the development of the strategies that enhance emotional recognition skills later in life.

### Limitations

Due to the constraints imposed by the COVID-19 pandemic, conducting data collection in a laboratory setting was not feasible and we administered an internet-based questionnaire via smartphones. We acknowledge that this methodological approach introduces a limitation to our study, as we did not control the exact size of facial stimuli across participants. While we recognize that this variable may have had an impact on the results, we believe that any potential influence would likely be consistent across our previous (Gori et al., 2021) and present studies. Furthermore, that another limitation of our study is the potential disparity in the duration and intensity of mask exposure between toddlers and adults. Indeed, legislation in Italy exempted children under the age of six from wearing masks, and toddlers typically spend more time at home compared to adults. Consequently, children may not have had as much interaction with individuals wearing facial masks as adults did. Nonetheless, the observed improvement in toddlers’ ability to recognize facial expressions with masks suggests that even limited and intermittent exposure can facilitate processes of learning and development. Finally, we acknowledge that the choice of static images of posed facial expressions might not fully reflect the capability of inferring emotions in real-life settings. Anyhow, again our results are significant when compared to data we originally collected using the same type of stimuli. Besides that, the choice of static images came from the need of implementing a test easy to be administered via internet, thus overcoming the social distancing rules that applied during the COVID-19 pandemic.

## Conclusion

In conclusion, our findings demonstrate that toddlers exhibit an enhanced ability to interpret emotions from masked facial expressions after one year of exposure to face masks. This observation provides a positive response to our initial inquiry regarding whether the altered availability of facial visual features during the COVID-19 pandemic might hinder or delay the development of emotion recognition skills in early childhood. Our results indicate that this is not the case; conversely, such development appears to undergo an acceleration. The resilient and adaptable nature of the brain, even under challenging conditions, likely fosters adaptation to enhance the face processing skills in young children, enabling them to successfully engage in social interactions.

## Data availability statement

The dataset presented in this study can be found online in Zenodo public repository at the following link: https://doi.org/10.5281/zenodo.11551299.

## Ethics statement

The studies involving humans were approved by Comitato Etico, ASL3 Genovese, Italy. The studies were conducted in accordance with the local legislation and institutional requirements. Written informed consent for participation in this study was provided by the participants’ legal guardians/next of kin.

## Author contributions

MG: Data curation, Writing – review & editing, Writing – original draft, Validation, Supervision, Methodology, Investigation, Funding acquisition, Conceptualization. LS: Investigation, Writing – review & editing, Writing – original draft, Validation, Conceptualization. MF: Writing – review & editing, Resources, Investigation. MBA: Writing – review & editing, Methodology, Investigation, Formal analysis, Data curation.

## References

[ref1] Amadeo M. B. Escelsior A. Amore M. Serafini G. Pereira da Silva B. Gori M. (2022). Face masks affect perception of happy faces in deaf people. Sci. Rep. 12:12424. doi: 10.1038/s41598-022-16138-x 35858937 PMC9298172

[ref2] Barrett L. F. Adolphs R. Marsella S. Martinez A. M. Pollak S. D. (2019). Emotional expressions reconsidered: challenges to inferring emotion from human facial movements. Psychol. Sci. Public Interest 20, 1–68. doi: 10.1177/1529100619832930, PMID: 31313636 PMC6640856

[ref3] Batty M. Taylor M. J. (2006). The development of emotional face processing during childhood. Dev. Sci. 9, 207–220. doi: 10.1111/j.1467-7687.2006.00480.x 16472321

[ref4] Bourke L. Lingwood J. Gallagher-Mitchell T. Lopez-Perez B. (2023). The effect of face mask wearing on language processing and emotion recognition in young children. J. Exp. Child Psychol. 226:105580. doi: 10.1016/j.jecp.2022.105580 36347070 PMC9637007

[ref5] Carbon C. C. Held M. J. Schutz A. (2022). Reading emotions in faces with and without masks is relatively independent of extended exposure and individual difference variables. Front. Psychol. 13:856971. doi: 10.3389/fpsyg.2022.856971, PMID: 35369259 PMC8967961

[ref6] Carnevali L. Gui A. Jones E. J. H. Farroni T. (2022). Face processing in early development: a systematic review of behavioral studies and considerations in times of COVID-19 pandemic. Front. Psychol. 13:778247. doi: 10.3389/fpsyg.2022.778247, PMID: 35250718 PMC8894249

[ref7] Carter C. S. Barch D. M. Gur R. Gur R. Pinkham A. Ochsner K. (2009). CNTRICS final task selection: social cognitive and affective neuroscience-based measures. Schizophr. Bull. 35, 153–162. doi: 10.1093/schbul/sbn157, PMID: 19011231 PMC2643972

[ref8] Chronaki G. Hadwin J. A. Garner M. Maurage P. Sonuga-Barke E. J. (2015). The development of emotion recognition from facial expressions and non-linguistic vocalizations during childhood. Br. J. Dev. Psychol. 33, 218–236. doi: 10.1111/bjdp.12075, PMID: 25492258

[ref8001] Chung M. S. Thomson D. M. (1995). Development of face recognition. Br J Psychol 86, 55–87. 7712070 10.1111/j.2044-8295.1995.tb02546.x

[ref9] Coppola C. P. (2014). Developmental Milestones. In: Eds. Coppola C. Kennedy Jr A. Scorpio R.. Pediatric Surgery. Springer, Cham: Springer. doi: 10.1007/978-3-319-04340-1_85

[ref10] Denham S. A. (1998). Emotional development in young children. New York: Guilford Press.

[ref11] Denham S. A. Bassett H. H. Zinsser K. Wyatt T. M. (2014). How preschoolers' social–emotional learning predicts their early school success: developing theory-promoting, competency-based assessments. Infant Child Dev. 23, 426–454. doi: 10.1002/icd.1840

[ref12] Denham S. A. Blair K. A. DeMulder E. Levitas J. Sawyer K. Auerbach-Major S.. (2003). Preschool emotional competence: pathway to social competence? Child Dev. 74, 238–256. doi: 10.1111/1467-8624.00533 12625448

[ref8002] De Sonneville L. M. Verschoor C. A. Njiokiktjien C. Op het Veld V. Toorenaar N. Vranken M.. (2022). Facial identity and facial emotions: speed, accuracy, and processing strategies in children and adults. J Clin Exp Neuropsychol. 24, 200-13.301. 10.1076/jcen.24.2.200.989 11992203

[ref13] Escelsior A. Amadeo M. B. Esposito D. Rosina A. Trabucco A. Inuggi A.. (2022). COVID-19 and psychiatric disorders: the impact of face masks in emotion recognition face masks and emotion recognition in psychiatry. Front. Psych. 13:932791. doi: 10.3389/fpsyt.2022.932791, PMID: 36238943 PMC9551300

[ref14] Fellows I. (2012). Deducer: a data analysis GUI for R. J. Stat. Softw. 49, 1–15. doi: 10.18637/jss.v049.i08

[ref15] Giordano K. Palmieri C. S. LaTourette R. Godoy K. M. Denicola G. Paulino H.. (2022). Face masks and emotion literacy in preschool children: implications during the COVID-19 pandemic. Early Child Educ. J. 52, 21–29. doi: 10.1007/s10643-022-01400-8 PMC9628515 36339523

[ref16] Gori M. Schiatti L. Amadeo M. B. (2021). Masking emotions: face masks impair how we read emotions. Front. Psychol. 12:669432. doi: 10.3389/fpsyg.2021.669432, PMID: 34113297 PMC8185341

[ref17] Grundmann F. Epstude K. Scheibe S. (2021). Face masks reduce emotion-recognition accuracy and perceived closeness. PLoS One 16:e0249792. doi: 10.1371/journal.pone.0249792, PMID: 33891614 PMC8064590

[ref18] Gur R. C. Sara R. Hagendoorn M. Marom O. Hughett P. Macy L.. (2002). A method for obtaining 3-dimensional facial expressions and its standardization for use in neurocognitive studies. J. Neurosci. Methods 115, 137–143. doi: 10.1016/S0165-0270(02)00006-7, PMID: 11992665

[ref19] Herba C. M. Landau S. Russell T. Ecker C. Phillips M. L. (2006). The development of emotion-processing in children: effects of age, emotion, and intensity. J. Child Psychol. Psychiatry 47, 1098–1106. doi: 10.1111/j.1469-7610.2006.01652.x, PMID: 17076748

[ref20] Herba C. Phillips M. (2004). Annotation: development of facial expression recognition from childhood to adolescence: behavioural and neurological perspectives. J. Child Psychol. Psychiatry 45, 1185–1198. doi: 10.1111/j.1469-7610.2004.00316.x, PMID: 15335339

[ref21] Kobayashi M. Macchi Cassia V. Kanazawa S. Yamaguchi M. K. Kakigi R. (2018). Perceptual narrowing towards adult faces is a cross-cultural phenomenon in infancy: a behavioral and near-infrared spectroscopy study with Japanese infants. Dev. Sci. 21:e12498. doi: 10.1111/desc.12498 27921339 PMC5763342

[ref22] Kohler C. G. Turner T. H. Gur R. E. Gur R. C. (2004). Recognition of facial emotions in neuropsychiatric disorders. CNS Spectr. 9, 267–274. doi: 10.1017/S1092852900009202 15048051

[ref23] Kolb B. Mychasiuk R. Muhammad A. Gibb R. (2013). Brain plasticity in the developing brain. Prog. Brain Res. 207, 35–64. doi: 10.1016/B978-0-444-63327-9.00005-9 24309250

[ref24] Macchi Cassia V. Bulf H. Quadrelli E. Proietti V. (2014). Age-related face processing bias in infancy: evidence of perceptual narrowing for adult faces. Dev. Psychobiol. 56, 238–248. doi: 10.1002/dev.21191, PMID: 24374735

[ref25] Marini M. Ansani A. Paglieri F. Caruana F. Viola M. (2021). The impact of facemasks on emotion recognition, trust attribution and re-identification. Sci. Rep. 11:5577. doi: 10.1038/s41598-021-84806-5, PMID: 33692417 PMC7970937

[ref26] McCrackin S. D. Capozzi F. Mayrand F. Ristic J. (2022). Face masks impair basic emotion recognition. Soc. Psychol. 54, 4–15. doi: 10.1027/1864-9335/a000470

[ref27] Miyazaki Y. Kamatani M. Suda T. Wakasugi K. Matsunaga K. Kawahara J. I. (2022). Effects of wearing a transparent face mask on perception of facial expressions. Iperception 13:20416695221105910. doi: 10.1177/20416695221105910, PMID: 35782828 PMC9243485

[ref28] Pavlova M. A. Carbon C. C. Coello Y. Sokolov A. A. Proverbio A. M. (2023). Editorial: impact of face covering on social cognition and interaction. Front. Neurosci. 17:1150604. doi: 10.3389/fnins.2023.1150604, PMID: 36895421 PMC9989270

[ref29] Pavlova M. A. Sokolov A. A. (2022). Reading covered faces. Cereb. Cortex 32, 249–265. doi: 10.1093/cercor/bhab311 34521105

[ref30] Pinkham A. E. Sasson N. J. Calkins M. E. Richard J. Hughett P. Gur R. E.. (2008). The other-race effect in face processing among African American and Caucasian individuals with schizophrenia. Am. J. Psychiatry 165, 639–645. doi: 10.1176/appi.ajp.2007.07101604, PMID: 18347000 PMC7413594

[ref31] Plate R. C. Wood A. Woodard K. Pollak S. D. (2019). Probabilistic learning of emotion categories. J. Exp. Psychol. Gen. 148, 1814–1827. doi: 10.1037/xge0000529, PMID: 30570327 PMC6586538

[ref32] R Core Team R. R: a language and environment for statistical computing. (2013).

[ref33] Rinck M. Primbs M. A. Verpaalen I. A. Bijlstra G. (2022). Face masks impair facial emotion recognition and induce specific emotion confusions. Cogn. Res. 7:83. doi: 10.1186/s41235-022-00430-5 PMC9444085 36065042

[ref34] Ruba A. L. Pollak S. D. (2020). Children's emotion inferences from masked faces: implications for social interactions during COVID-19. PLoS One 15:e0243708. doi: 10.1371/journal.pone.0243708, PMID: 33362251 PMC7757816

[ref35] Thomas K. M. Drevets W. C. Whalen P. J. Eccard C. H. Dahl R. E. Ryan N. D.. (2001). Amygdala response to facial expressions in children and adults. Biol. Psychiatry 49, 309–316. doi: 10.1016/S0006-3223(00)01066-0 11239901

[ref36] Tonks J. Williams W. H. Frampton I. Yates P. Slater A. (2007). Assessing emotion recognition in 9-15-years olds: preliminary analysis of abilities in reading emotion from faces, voices and eyes. Brain Inj. 21, 623–629. doi: 10.1080/02699050701426865, PMID: 17577713

[ref37] Ventura M. Palmisano A. Innamorato F. Tedesco G. Manippa V. Caffò A. O.. (2023). Face memory and facial expression recognition are both affected by wearing disposable surgical face masks. Cogn. Process. 24, 43–57. doi: 10.1007/s10339-022-01112-2, PMID: 36242672 PMC9568966

[ref38] Wheeler B Torchiano M. lmPerm: permutation tests for linear models; (2010). R package version.

[ref39] World Health Organization. Advice on the use of masks for children in the community in the context of COVID-19. Annex to the advice on the use of masks in the context of COVID-19. (2020). Available at: https://www.who.int/publications/i/item/WHO-2019-nCoV-IPC_Masks-Children-2020.1.

